# Dynamic–Attentive Pooling Networks: A Hybrid Lightweight Deep Model for Lung Cancer Classification

**DOI:** 10.3390/jimaging11080283

**Published:** 2025-08-21

**Authors:** Williams Ayivi, Xiaoling Zhang, Wisdom Xornam Ativi, Francis Sam, Franck A. P. Kouassi

**Affiliations:** 1 School of Information and Communication Engineering, University of Electronic Science and Technology of China, Chengdu 611731, China; 2 Department of Information Systems and Operations Management, Vienna University of Economics and Business, 1020 Vienna, Austria; 3 School of Computer Science and Technology, University of Electronic Science and Technology of China, Chengdu 611731, China; ativiw@std.uestc.edu.cn (W.X.A.); franckarmand@std.uestc.edu.cn (F.A.P.K.); 4 School of Information and Software Engineering, University of Electronic Science and Technology of China, Chengdu 610054, China; fransamang@std.uestc.edu.cn

**Keywords:** deep learning, ResNet50, second-order pooling, squeeze-and-excitation attention, dynamic feature enhancement

## Abstract

Lung cancer is one of the leading causes of cancer-related mortality worldwide. The diagnosis of this disease remains a challenge due to the subtle and ambiguous nature of early-stage symptoms and imaging findings. Deep learning approaches, specifically Convolutional Neural Networks (CNNs), have significantly advanced medical image analysis. However, conventional architectures such as ResNet50 that rely on first-order pooling often fall short. This study aims to overcome the limitations of CNNs in lung cancer classification by proposing a novel and dynamic model named LungSE-SOP. The model is based on Second-Order Pooling (SOP) and Squeeze-and-Excitation Networks (SENet) within a ResNet50 backbone to improve feature representation and class separation. A novel Dynamic Feature Enhancement (DFE) module is also introduced, which dynamically adjusts the flow of information through SOP and SENet blocks based on learned importance scores. The model was trained using a publicly available IQ-OTH/NCCD lung cancer dataset. The performance of the model was assessed using various metrics, including the accuracy, precision, recall, F1-score, ROC curves, and confidence intervals. For multiclass tumor classification, our model achieved 98.6% accuracy for benign, 98.7% for malignant, and 99.9% for normal cases. Corresponding F1-scores were 99.2%, 99.8%, and 99.9%, respectively, reflecting the model’s high precision and recall across all tumor types and its strong potential for clinical deployment.

## 1. Introduction

Lung cancer remains one of the leading causes of cancer-related mortality worldwide, with 2.21 million new cases and 1.8 million deaths recorded in 2020, according to the World Health Organization (WHO) [1]. Non-Small-Cell Lung Cancer (NSCLC) accounts for nearly 85% of all lung cancer cases, and early detection significantly improves survival rates [2]. The five-year survival rate for early-stage NSCLC can exceed 60%, whereas late-stage detection drastically reduces survival to less than 5% [1]. Low-dose computed tomography (LDCT) screening has proven effective in reducing lung cancer mortality by 20% in high-risk individuals [2]. However, a manual CT scan interpretation by radiologists remains subjective, time-intensive, and prone to inter-observer variability, leading to misdiagnoses, false positives, and delayed treatment [3,4]. These challenges necessitate the development of automated, AI-driven diagnostic solutions that can enhance the accuracy, efficiency, and reproducibility of lung cancer classification.

While traditional radiological methods remain the gold standard, Computer-Aided Diagnosis (CAD) systems using machine learning have emerged to assist radiologists [5]. However, early CAD systems rely on handcrafted features and statistical models, which often struggle with complex tumor morphologies. In contrast, deep learning (DL), particularly Convolutional Neural Networks (CNNs), have revolutionized medical imaging by automatically extracting hierarchical features from CT scans, significantly enhancing the diagnostic accuracy and reducing human variability [6].

CNN-based architectures alone such as ResNet50, VGG16, and DenseNet have demonstrated exceptional accuracy in lung cancer classification tasks [1,7]. However, these models still suffer from critical limitations, particularly in feature extraction, generalization to clinical data, and computational efficiency [8,9,10].

Traditional CNNs rely on first-order pooling operations (e.g., max pooling or average pooling) to reduce feature map dimensions [11,12]. However, these techniques often lose meaningful spatial relationships in lung tumor textures, making it challenging to differentiate between malignant and benign lesions. While Squeeze-and-Excitation Networks (SENet) have been introduced to improve channel-wise attention, existing models apply static recalibration mechanisms, meaning all feature maps receive equal importance across layers, leading to unnecessary computations and reduced efficiency [13,14]. Current DL models apply uniform feature enhancement strategies, failing to prioritize diagnostically relevant features while suppressing background noise dynamically. This hinders the model’s ability to generalize across diverse CT scans, often resulting in overfitting to training data and a poor real-world performance [1].

Recent research has emphasized the limitations of static improvement and argued for dynamic, context-aware feature selection. Kalkan et al. showed that combining InceptionResNetV2 with UNet-based segmentation improves the classification and localization performance, attaining 98.5% accuracy and a 95.3% Jaccard index on complicated CT datasets. Their findings back up the assumption that hybrid and multi-task networks improve the interpretability and clinical value [15]. Furthermore, Ekinci et al. [16] shown that attention-enhanced CNNs and second-order pooling considerably improve the lung cancer classification by better capturing inter-channel dependencies and textural variability. Their findings establish a convincing case for second-order statistical representations in imaging applications. Guzel et al. [17] found that networks incorporating adaptive pooling, SE blocks, and residual fusion produced cutting-edge results across a wide range of imaging procedures and scanner setups. Their findings highlight the need to establish feature selection techniques that account for intra-class variability and image resolution. Similarly, Asuroglu et al. [15] advocated for lightweight but expressive models designed for therapeutic use. They argued for architectures that give a high level of performance at a cheap computational cost which are essential for real-time diagnostic systems in low-resource environments.

These current findings emphasize the necessity of hybrid designs that integrate residual feature extraction, dynamic attention, and statistical pooling. This convergence emphasizes the research vacuum that our study aims to fill: the need for a compact, adaptive, and context-aware model that may dynamically improve essential diagnostic clues while suppressing superfluous information.

While ResNet50 has been widely adopted for lung cancer classification due to its strong feature extraction capabilities, it struggles to fully capture fine-grained tumor textures, often leading to misclassification. The integration of SENet improves channel-wise recalibration, but its uniform application across all layers introduces computational overhead and may amplify irrelevant features. Furthermore, current hybrid models lack dynamic feature selection, applying feature enhancement mechanisms indiscriminately across feature maps without considering the diagnostic relevance.

To address these limitations, we introduce dynamic–attentive pooling networks, a deep learning model that dynamically activates feature recalibration using a novel Dynamic Feature Enhancement (DFE) module. Unlike existing CNN-based classifiers that uniformly apply SOP and SENet, DFE learns when and where to apply these enhancements, ensuring that only diagnostically relevant features are emphasized, while redundant computations are minimized.

The primary contributions of this research are as follows:

1. Our model integrates SOP, SENet, and DFE to improve lung cancer classification using CT images. It uses a dynamically activated or bypassed DFE module, allowing for better feature selection and optimal classification. This model overcomes limitations of first-order pooling in CNNs.
2. We validated the model’s generalizability using an augmented dataset and raw non-augmented clinical data. The DFE module ensures the selective application of SOP and SENet, while advanced augmentation strategies introduce dataset diversity, making the model adaptable to diverse clinical settings.
3. The model performs well in lung cancer classification, utilizing second-order statistical learning, adaptive feature selection, and dynamic feature recalibration for superior accuracy and reliability.

To the best of our knowledge, this study is among the first to integrate SOP, SENet, and an adaptive feature selection mechanism DFE within a ResNet50 framework for lung cancer classification. By introducing dynamic decision making into CNN-based medical imaging models, our model marks a significant advancement in AI-driven healthcare technologies, ensuring a balance between the diagnostic performance and computational efficiency, making it a strong candidate for real-world clinical deployment.

The rest of this work is organized into sections as follows: Section 2 delineates the methodology, emphasizing the augmented ResNet50 model that integrates squeeze-and-excitation attention processes and second-order pooling techniques. Section 3 presents the experimental findings and compares them to recent methods on cutting-edge datasets. Lastly, Section 4 provides the conclusion and explores possible directions for future research.

## 2. Methodology

In this section, we present a dynamic–attentive pooling network, a deep learning model that integrates SOP and SENet into the ResNet50 architecture to enhance the lung cancer classification accuracy. The proposed approach optimizes feature representation, addresses class imbalance, and improves model robustness against variations in CT scan quality. By selectively applying SENet and SOP through an adaptive DFE module, the network ensures that feature recalibration and higher-order pooling are applied only when necessary, thereby enhancing both the classification performance and computational efficiency.

### 2.1. Problem Statement

Lung cancer remains one of the leading causes of cancer-related mortality worldwide, highlighting the urgent need for highly reliable and generalizable deep learning models for early detection and classification. Traditional CNN-based models, such as ResNet50 and its pretrained variants ResNet 50(*), exhibit significant limitations in feature representation, classification robustness, and generalization to real-world clinical datasets. These challenges primarily stem from their reliance on first-order pooling mechanisms, which fail to capture complex spatial dependencies and fine-grained tumor characteristics, often leading to increased false positives and false negatives—a critical issue in clinical diagnostics.

To address these limitations, we propose a DL-based model that integrates SOP and SENet within an adaptive DFE framework. Unlike previous approaches that apply feature recalibration and pooling mechanisms uniformly across all layers, our method dynamically activates SENet and SOP only when necessary, optimizing both the classification performance and computational efficiency. Additionally, we introduce a two-phase evaluation strategy, wherein the model is first trained and tested on an augmented dataset before undergoing validation on raw non-augmented clinical data. This approach significantly enhances the model robustness, improves generalization to real-world applications, and mitigates overfitting risks associated with limited dataset diversity, ensuring that our model remains a clinically viable and efficient AI-driven diagnostic tool.

### 2.2. Baseline Architecture

Deep Learning algorithms, such as ResNet, have demonstrated inconsistent efficacy in extracting significant characteristics from medical images such as CT, resulting in the accurate and efficient diagnoses of various diseases. The residual block is the basic building block of a residual network. One of the models developed by He et al. in 2016 was the deep residual network, or ResNet [18]. This network was designed to address the limitations of traditional deep learning training, which often requires extensive time, and is also constrained by the number of layers it can effectively handle. ResNet’s complexity can be simplified by using shortcuts to skip connections. The ResNets’ model outperforms other designs by maintaining a high performance as the network complexity rises. It also reduces the computation time and improves the network training efficiency.

The ResNet model skips connections on two to three layers, including ReLU, and uses batch normalization in the architecture [18]. According to [18], the ResNet model outperformed other models in image classification, indicating effective feature extraction by ResNet.

**Figure 1:**
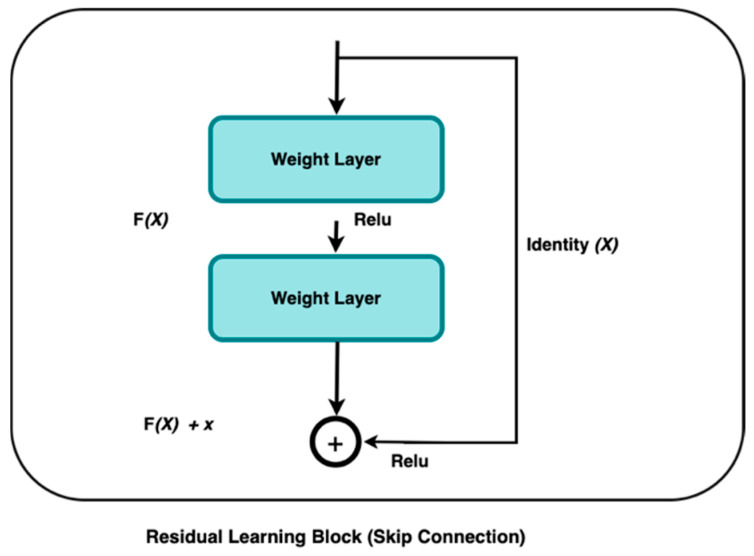
Residual learning block.

He et al. [18] apply residual learning to multiple layers. The ResNet residual block in Figure 1 is defined as follows:

(1) $$s=F\left(x,W+x\right)$$

where x is the input layer, y is the output layer, and the residual map represents the F function.

This research used ResNet50 as our baseline model due to its ability to extract compact yet expressive features. Residual learning is fundamental to this architecture, as described by the authors in [18]. The residual block’s operation can be expressed as:

(2) $$y=F\left(x,W\right)+x$$

Here, x is the residual block, y produces, and $F\left(x,W\right)$ is the residual function that represents the transformation applied to x with weights W.

The addition of x to the output of the residual function $F\left(x,W\right)$ can help to overcome the problem of the vanishing gradient, allowing for the effective training of deeper networks. However, ResNet50 relies on first-order pooling operations, such as max pooling and average pooling, which capture only localized spatial information while discarding intricate feature relationships [19]. This limitation can lead to suboptimal tumor classification, particularly in cases where subtle textural differences distinguish benign from malignant regions.

To combat the limitations of first-order pooling in ResNet50, we integrate Second-Order Pooling (SOP) and Squeeze-and-Excitation Networks (SENet) to enhance the feature representation and classification performance. SOP enables the model to capture complex spatial dependencies and global feature interactions, preserving intricate tumor characteristics that would otherwise be lost in traditional pooling operations. Simultaneously, SENet dynamically recalibrates channel-wise feature importance, ensuring that diagnostically relevant patterns receive greater emphasis while suppressing irrelevant background noise. This synergistic integration strengthens the model’s ability to extract, prioritize, and leverage critical tumor features, leading to superior discriminative power, robustness, and diagnostic accuracy in lung cancer classification.

### 2.3. Squeeze and Excitation Attention (SENet)

Neural network models employ the squeeze operation to reduce the dimensionality of feature maps by combining their spatial elements into a single scalar value [13]. The network’s ability to recognize and respond to global patterns within the input data is due to its creation of a global receptive field. The excitation phase operates analogously to the gating mechanism utilized in recurrent neural networks. This method enables the network to concentrate on the most pertinent aspects of the input data by enhancing or diminishing certain traits according to their relevance to the job at hand. The squeezing and excitation actions together allow a neural network to modulate the importance of various channels, hence improving its capacity to identify intricate patterns and relationships within the data [20].

A neural network can more effectively learn intricate patterns and relationships in the data by dynamically adjusting the weight of various channels thanks to squeeze and excitation operations [21]. In order to explicitly model the correlation between feature channels, it uses parameters to generate weights for each feature channel, which it then learns.

Squeeze-and-Excitation (SE) blocks were introduced to improve channel-wise feature recalibration by dynamically assigning importance weights to different feature maps [13]. In standard CNN architectures, all channels contribute equally to final feature maps, but some channels are more informative than others, particularly in medical imaging where tumor regions are sparsely distributed.

The SE block consists of three operations: Squeeze, Excitation, and Recalibration. Given a feature map ${X\in R}^{C\times H\times W}$, the SE block computes a per-channel attention score based on the global context.

#### 2.3.1. Squeeze (Global Average Pooling)

The spatial information is compressed into a channel descriptor vector using Global Average Pooling (GAP):

(3) $$z_{c}=\frac{1}{H\times W}\sum_{i=1}^{H}\sum_{j=1}^{W}X_{c}\left(i,j\right)$$

where $z_{c}$ is the aggregated global feature response for each channel c.

#### 2.3.2. Excitation (Channel-Wise Dependencies via Fully Connected Layers)

The aggregated vector is passed through two fully connected (FC) layers with activation functions:

(4) $$s=\sigma (W_{2}\delta \left(W_{1}z\right))$$

where:

- $W_{1}\in R\frac{c}{r}\times c$ ensures dimentionality reduction (r is the reduction ratio).
- $\delta \left(\cdot \right)$ is the ReLU activation function.
- $W_{2}\in R^{c}\times \;\frac{c}{r}$ expands back to full dimensions.
- $\sigma \left(\cdot \right)$ is the sigmoid activation, normalizing values between 0 and 1.

#### 2.3.3. Recalibration (Multiplicative Attention on Channels)

The output is used as a set of channel-wise attention scores, refining the feature representation:

(5) $$\tilde{X_{c}}=s_{c}\cdot X_{c}$$

where $\tilde{X_{c}}$ represents the recalibrated feature map with enhanced attention on meaningful lung features.

**Figure 2:**
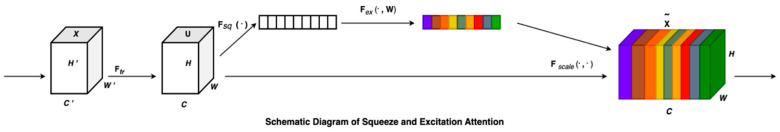
SENet architecture.

By integrating SENet into ResNet50, the model is able to suppress irrelevant background information and focus on key pathological features in CT scans. This helps boost the classification performance by ensuring that feature maps relevant to cancerous regions receive higher activation importance. Figure 2 provides the graphical representation of the SENet block.

For every transformation, $F_{tr}$, that maps the input $X\left(X\in R^{{\left(H^{\prime }\times W^{\prime }\times C\right)}^{\prime }}\right)$ to the feature map U($U\in R^{\left(H\times W\times C\right)})$, an SE block can be reconstructed to recalibrate the features. The feature U first undergoes a squeeze operation $F_{sq}$ followed by excitation. Finally, the calibration is applied through $F_{scale}$ where the excitation weights are multiplied individually with each preceding feature channel. This process fine-tunes the importance of each feature channel by recalibrating the original characteristics in the channel dimension, leading to several key benefits:

- SE blocks enhance critical channels that focus on malignant regions, suppressing less relevant background noise.
- The non-linear transformation in SE blocks improves the ability to distinguish between benign and malignant cases, particularly when tumors are small and difficult to detect.
- SE-based feature weighting makes the model more generalizable across multiple datasets, reducing overfitting to dataset-specific intensity distributions.

### 2.4. Second-Order Pooling (SOP)

**Figure 3:**
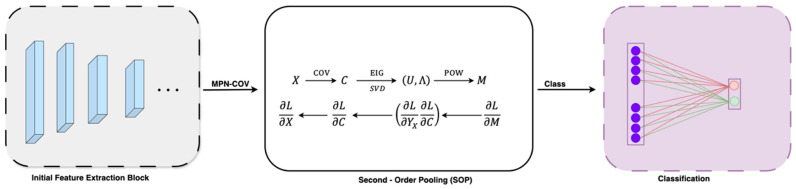
Second-order pooling.

In deep networks, second-order pooling, as shown in Figure 3, proves to be more powerful and discriminative than first-order pooling when it comes to feature classification [22,23]. Over the last decade, global second-order covariance representations, normalized using matrix power, have been widely applied to various large-scale visual recognition challenges. Building on this, we propose utilizing covariance merging to extract second-order statistical features from lung cancer CT images to enhance the model’s classification accuracy. Pooling operations in deep learning aim to reduce spatial dimensions while retaining essential feature information. Traditional pooling methods such as Max Pooling and Average Pooling aggregate first-order statistics (mean or max activations), which can lead to a loss of structural information [11]. In contrast, SOP captures higher-order statistics, leading to a more expressive and discriminative feature representation. This approach offers several advantages:

- It captures pairwise feature correlations, preserving texture variations in tumor regions for improved classification.
- Covariance normalization enhances the scale invariance, making the model robust to intensity variations in CT scans.
- Eigenvalue normalization stabilizes the feature distribution, preventing extreme activation magnitudes that could lead to unstable gradient updates.

The detailed second-order pooling model is outlined below.

#### 2.4.1. Covariance Normalization

In SOP, the covariance matrix of the feature maps is used as a representation of global features. Given an input feature ${map\;X\in R}^{C\times H\times W}$, where C is the number of channels and H,W are spatial dimensions, SOP computes the second-order feature representation as:

(6) $$S\;=\;\left(\frac{1}{N}\right)\sum_{i=1}^{N}\left(x_{i}-\mu \right){\left(x_{i}-\mu \right)}^{T}$$

(7) $$F=\varphi \left(X;W,b\right)$$

Here, W and b denote the weights and biases, respectively. The function φ is derived from a CNN stream composed of convolutional layers, non-linear units, ReLU, and max-pooling layers. To integrate the covariance matrix C into the deep learning model, the output feature map, ${X\in R}^{W\times H\times C}$, is first transformed into a ddd-dimensional feature matrix ${F\in R}^{d\times N}$ where N=H×W represents the total number of features.

(8) $$C={F\bar{I}F}^{T}$$

Here, $\bar{I}=\frac{1}{N}\left(I-\frac{1}{N}{JJ}^{T}\right)I$ represents an N×N identity matrix, where J is a *d*-dimensional vector consisting entirely of ones, and TTT denotes the transpose of the matrix. The singular value decomposition (SVD) or eigenvalue decomposition (EIG) of the symmetric positive semidefinite (SPD) covariance matrix C is given by the following formula:

(9) $$CC\to \left(U,\wedge \right),\;C=U\wedge U^{T}$$

The matrix λ, denoted as $diag\left(\lambda_{1},\ldots ,\;\lambda_{d}\right)$, represents a diagonal matrix where $\lambda_{1}$(i=1,…,d) are eigenvalues, ordered from largest to smallest. The matrix $U=\;[u_{1},\ldots ,u_{d}$] is orthogonal, and each uiu_iui is an eigenvector that corresponds to the eigenvalue $\lambda_{i}$.

The power of the covariance matrix C is computed using the eigenvalues and the orthogonal matrix derived from either the EIG or the SVD.

(10) $$\left(U,\wedge \right)\to M,M=C^{\alpha }=U\Phi \left(\wedge \right)U^{T}$$

In this instance, $\Phi \left(\wedge \right)$= $diag\left(\lambda_{1},\ldots ,\;\lambda_{d}\right)$ and the exponent α is a positive real number. Experiments prove that the covariance matrix has the best effect when α is 0.5.

#### 2.4.2. Covariance Normalization Acceleration

As mentioned earlier, both SVD and EIG play essential roles in second-order computation. However, executing these operations on a GPU is typically slower compared to a CPU. To address this, the Newton–Schulz iteration method can be applied to speed up the process of covariance normalization, thereby enhancing its efficiency for GPU-based computations. Finally, the linked iteration may be described in the following format:

(11) $$Y_{n}=\frac{1}{2}Y_{n-1}\left(3I-M_{n-1}Y_{n-1}\right)$$

(12) $$M_{n}=\frac{1}{2}\left(3I-M_{n-1}Y_{n-1}\right)M_{n-1}$$

The optimum solution can be achieved with a minimal number of iterations, specifically five in this case. To ensure the Newton–Schulz iteration converges effectively, the following equation has been formulated:

(13) $$\hat{C}=\frac{1}{tr\left(C\right)}C$$

In the equation mentioned earlier,$\;tr\left(\cdot \right)$ represents the trace of the matrix. Additionally, the change in data magnitude introduced by this equation can be adjusted using the subsequent post-compensation formula:

(14) $$M=\sqrt{tr\left(C\right)Y_{N}}$$

The pseudocode of the SOP algorithm is presented in Algorithm 1.
**Algorithm 1: Second-Order Pooling (SOP) with Newton–Schulz Iteration****Require**: Feature map $X\in R^{C\times H\times W}$ **Ensure**: Second-order pooled features $M\in R^{C\times C}$1.  F = Reshape (X, [C,H×W])      ▷ *Flatten spatial dimensions*2.  N=H×W3.  $\bar{I}=\frac{1}{N}(I-\frac{1}{N}JJ^{T})$              ▷ *Centering matrix*4.  $C=F\bar{I}F^{T}$                  ▷ *Covariance matrix*5.  $\hat{C}\;=\frac{c}{tr\left(c\right)}\;$             ▷ *Normalize for convergence*6.  $Y_{0}=\;\hat{C},\;M_{0}=I$             ▷ *Initialize Newton–Schulz*7.  **for** n=1 to 5 **do**          ▷ *5 iterations for convergence*8.    $Y_{n}=\frac{1}{2}Y_{n-1}(3I-M_{n-1}Y_{n-1})$9.    $M_{n}=\frac{1}{2}\left(3I-M_{n-1}Y_{n-1}\right)M_{n-1}$10.**end for**11.$M\;=\;\surd tr(C)\cdot Y_{5}$              ▷ *Post-compensation*12.**return** MDFE Model

### 2.5. The Development and Description of the Model

The efficacy of DL models in establishing robust lung cancer classification frameworks hinges on the extraction of highly discriminative features from CT scan inputs. In lung CT imaging, irrelevant background information can obscure critical tumor features, hampering the model performance. To enhance the diagnostic accuracy, our model integrates several key advancements, combining DFE modules with SOP and SENet. These enhancements work synergistically to improve feature extraction, refine classification robustness, and enhance generalizability across diverse imaging conditions.

Our model employs a ResNet50 backbone with an optimized hierarchical feature extraction mechanism. The architecture begins with a 7 × 7 convolutional layer, followed by max pooling, which extracts primary image features such as edges, textures, and contrast variations in lung CT scans. To ensure adaptive feature recalibration at critical stages, a DFE module is strategically introduced before the first SOP block. This module dynamically assesses feature importance and selectively activates SOP, allowing the model to capture higher-order spatial correlations only when necessary, reducing redundant computations.

Following this, the extracted feature maps undergo progressive refinement through three hierarchical stages of Res-Blocks, structured as 3, 4, and 6 layers, respectively. These layers enable deep feature learning by preserving the gradient flow while extracting increasingly complex tumor characteristics. Before entering the final SENet block, a second DFE module is applied to modulate channel recalibration dynamically, ensuring SENet operates only on the most diagnostically relevant feature channels. This adaptive recalibration strategy prevents the unnecessary processing of uninformative features, balancing computational efficiency with enhanced feature prioritization.

In the classification phase, an SE Attention Block further refines the extracted features before average pooling condenses the feature maps. A final SOP block enhances the feature space by capturing fine-grained tumor textures and global feature relationships. The classification head consists of a fully connected layer, transforming the refined features into class logits, followed by a softmax activation function for final classification. This structured approach ensures high diagnostic accuracy and improved robustness in distinguishing between benign, malignant, and normal lung tissues.

The integration of SOP at both the feature extraction and classification stages represents a significant innovation in our model, enabling the model to leverage intricate spatial dependencies for improved classification. SENet further enhances the model’s ability to suppress background noise and amplify diagnostically relevant features, leading to superior sensitivity and specificity in lung cancer detection.

Moreover, the model is optimized for computational efficiency, ensuring that while the integration of DFE modules, SOP, and SENet introduces additional computations, the model remains feasible for real-time clinical deployment. The adaptive feature selection strategy of DFE significantly reduces redundant processing, making the model suitable for hospital-grade AI-assisted diagnostic systems. While training requires additional GPU memory due to higher-order feature computations, inference remains computationally efficient, facilitating real-time diagnostic applications.

To further enhance real-world deployment, the model incorporates efficient layer parameterization, optimized matrix factorization in SOP, and batch normalization techniques to reduce computational redundancy.

Basically, our model is defined as:

(15) $$\hat{y}=softmax(W_{c}\cdot (\sigma \left(W_{2}\delta \left(W_{1}s_{c}\right)\right)\cdot f_{DFE}(S^{\frac{1}{2}}))+b$$

where:

- $S^{\frac{1}{2}}$ represents the normalized second-order pooled feature matrix that captures higher-order spatial correlations in tumor textures.
- $s_{c}=\frac{1}{H\times W}\sum_{i=1}^{H}\sum_{j=1}^{W}H_{L}\left(i,j\right)$ is the global descriptor extracted through Global Average Pooling (GAP) from the final feature map $H_{L}$.
- $e_{c}=\sigma \left(W_{2}\delta \left(W_{1}s_{c}\right)\right)$ is the channel-wise recalibration weight which is computed using fully connected layers where:
  ▪ $W_{1}$ and $W_{2}$ are learnable parameters.
  ▪ $\delta \left(\cdot \right)$ is the ReLU activation function.
  ▪ $\sigma \left(\cdot \right)$ represents the sigmoid function that ensures values remain in [0, 1].
- $H_{L}^{\prime }=e_{c}\cdot H_{L}$ represents the feature-enhanced output, where SENet selectively amplifies important tumor features.
- $W_{c}$ and b are fully connected layer parameters, transforming recalibrated features into final classification logits.
- $\hat{y}$ is the predicted probability distribution over lung cancer classes that are obtained using the softmax function.

To obtain a reliable and faithful model, the second-order pooling operation $S^{\frac{1}{2}}$ enables the model to retain global spatial correlations across lung CT scan features, enhancing its ability to distinguish fine-grained tumor textures.

The incorporation of SENet further improves model reliability by dynamically adjusting channel-wise importance, ensuring that diagnostic features receive greater emphasis, while non-relevant features are suppressed.

**Figure 4:**
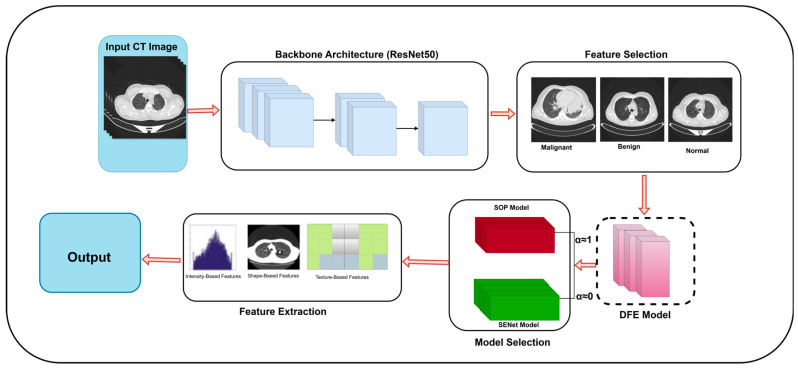
Proposed methodology pipeline.

**Figure 5:**
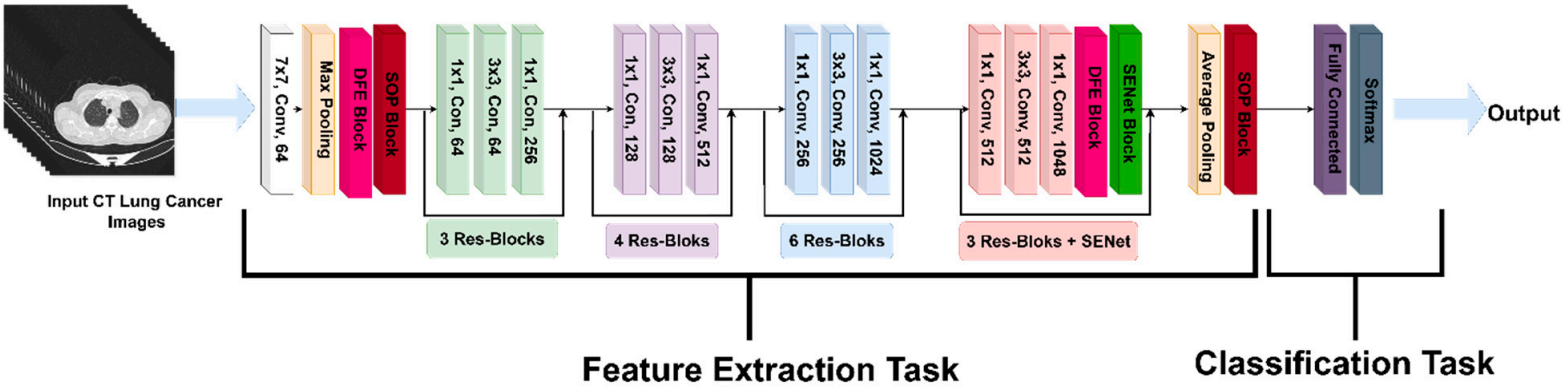
Lung SE-SOP architecture.

To further enhance the feature selection and computational efficiency, a DFE module is introduced, allowing the model to adaptively decide when SOP and SENet should be activated. Instead of uniformly applying feature recalibration, DFE selectively enables or bypasses SOP and SENet based on learned feature importance scores. Figure 4 presents the proposed methodology pipeline and Figure 5 is the main architecture proposed for this research. Algorithm 2 below represents the pseudocode of our DFE module.

The DFE module modifies the feature selection function as follows:

(16) $$f_{DFE}{(S}^{\frac{1}{2}})=\alpha \cdot S^{\frac{1}{2}}+(1-\alpha )\cdot H_{L}^{\prime }$$

where:

- α= sigmoid ${(W}_{d}\cdot s_{c})$ is an attention-based gating function that dynamically determines whether SOP should be applied or bypassed.
- $W_{d}$ is a learnable weight matrix that decides the influence of SOP dynamically.
- When α≈1, the model prioritizes second-order pooling, preserving global feature interactions for complex tumor structures.
- When α≈0, the model relies on SENet-based recalibration, preventing unnecessary SOP computations and reducing computational overhead.

While traditional CNN architectures rely on first-order pooling methods, our model utilizes SOP, SENet, and DFE to refine the extracted feature maps, achieving a more discriminative and robust classification pipeline. The DFE module introduces an adaptive decision-making strategy, ensuring that only relevant spatial and channel-wise transformations are applied, thereby balancing a high diagnostic performance with computational efficiency.

This approach effectively reduces misclassification errors and improves generalization, making the model a hybrid–lightweight deep learning framework. The model’s adaptive nature ensures that it remains suitable for real-time clinical applications, minimizing computational redundancy while maximizing sensitivity and specificity in lung cancer detection.
**Algorithm 2: Dynamic Feature Enhancement (DFE) Module for Adaptive Feature Selection****Require**: Feature map $H_{L}\;\in \;R^{C\times H\times W}$ from ResNet stage, Importance threshold τ = 0.5 **Ensure**: Adaptively enhanced features $F_{enhanced}$ with selective SOP/SENet application1.  **Parameters**: Learnable weight matrix $W_{d}\;\in \;R^{C\times C}$, bias $b_{d}\;\in \;R^{C}$2.  $s_{c}\;=\;\frac{1}{H\times W}\;\;\Sigma_{i=1}^{H}\;\Sigma_{j=1}^{W}\;H_{L}(i,j$)              ▷ *Global Average Pooling*3.  $z=W_{d}\cdot s_{c}+b_{d}\;$                   ▷ *Linear transformation*4.  α=σ(z)                ▷ *Sigmoid activation for gating* $\in \left[0,1\right]$5.  **if** α>τ **then**         ▷ *High importance: Apply SOP for complex features*6.    S= SecondOrderPooling $\left(H_{L}\right)$        ▷ *Compute covariance matrix*7.    $S^{1/2}=$ MatrixSquareRoot(S)          ▷ *Newton–Schulz iteration*8.    $F_{SOP}=$ Flatten $\left(S^{1/2}\right)$9.    $F_{enhanced}=\alpha \cdot F_{SOP}+(1-\alpha )\cdot H_{L}$            ▷ *Weighted combination*10.**else**          ▷ *Low importance: Apply SENet for channel recalibration*11.  $e_{c}\;=\;S$ ENetBlock $\left(H_{L}\right)$            ▷ *Channel attention weights*12.  $H_{L}^{\prime }=e_{c}⊙H_{L}$                 ▷ *Channel-wise multiplication*13.  $F_{enhanced}=\;(1-\alpha )\cdot H_{L}^{\prime }\;+\alpha \cdot H_{L}$14.**end if**15.Compute $R_{DFE}={\left\|\alpha -0.5\right\|}_{2}$        ▷ *Regularization for balanced selection*16.**return** $F_{enhanced}\cdot \;R_{DFE}$


### 2.6. Dataset

#### 2.6.1. IQ-OTH/NCCD Dataset and Preprocessing

The experiments in this study utilize the Iraq-Oncology Teaching Hospital/National Center for Cancer Diseases (IQ-OTH/NCCD) lung cancer dataset [24], a publicly available and well-annotated dataset that serves as a valuable resource for advancing lung cancer detection models. Collected over a three-month period in 2019 from the Iraq-Oncology Teaching Hospital and the National Center for Cancer Diseases, the dataset comprises high-resolution CT scans, annotated by an expert team of radiologists and oncologists. This ensures high-quality and clinically relevant imaging data suitable for deep learning-based analysis.

**Figure 6:**
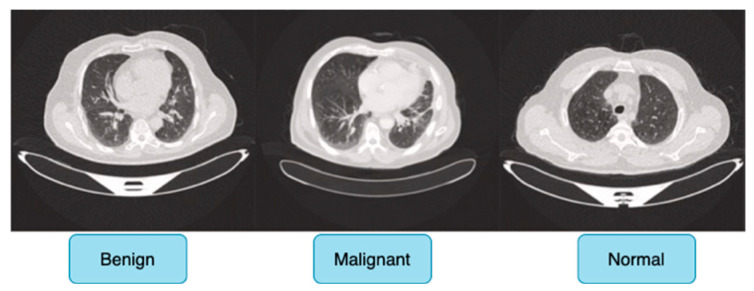
IQ-OTH/NCCD lung cancer CT dataset.

The IQ-OTH/NCCD dataset comprises 1190 CT scan slices from 110 individuals with diverse demographic backgrounds, including age, gender, education level, place of residence, and lifestyle factors, and is categorized into three diagnostic groups: 40 malignant cases, 15 benign cases, and 55 normal (healthy) cases, ensuring a balanced and meaningful distribution for classification tasks. Figure 6 shows sample images for benign, malignant and normal lung cancer CT images from the dataset.

Originally, the dataset comprised 512 × 512 DICOM CT images; however, to enhance compatibility with deep learning frameworks while preserving medical fidelity, these images were converted to a lossless PNG format. This conversion retains essential radiological features, ensuring the dataset remains suitable for robust feature extraction in deep learning-based cancer detection models. The dataset is publicly accessible via the Kaggle platform, promoting reproducibility and further research advancements [24].

All images were resized to 224 × 224 pixels to match the input requirements of ResNet50, ensuring computational efficiency without a significant loss of detail, while pixel intensity values were normalized to [0, 1] to enhance the network convergence and performance stability. The dataset was then split into training (70%), validation (15%), and test (15%) sets using stratified sampling to maintain a proportional representation of each class, preventing data leakage and ensuring fair model evaluation.

#### 2.6.2. Data Augmentation Technique

To enhance the training of the deep CNN architecture, extra samples were created for each class through the application of data augmentation techniques on the original set of 1097 samples. This strategy not only expanded the dataset, but also improved model robustness and helped mitigate the risk of overfitting. A commonly employed technique for artificially expanding a dataset is termed data augmentation. It involves performing several transformations along both horizontal and vertical axes, such as translation, rotation, shearing, mirroring, cropping, and flipping. Six augmentation techniques—rotation, brightness modification, zooming, width shifting, height shifting, and horizontal flipping—were applied in this study to assist in balancing the dataset’s class distribution.

Table 1 represents the total number of datasets in the IQ-OTH/NCCD dataset before and after it has undergone the data augmentation technique. Additionally, to address the class imbalance, resampling methods combined with transformations like Gaussian noise and Salt-and-Pepper noise were applied. These augmentations aimed to generate a more complex and diverse dataset, ultimately improving the model’s accuracy and generalizability.

#### 2.6.3. Evaluation Metrics

Quantitative metrics, referred to as evaluation measures, are essential for assessing the performance of deep learning models. These metrics are crucial for comparing the effectiveness of different models or algorithms on a specific task, identifying successful strategies, and highlighting areas that require further improvement. In this study, the evaluation metrics utilized include recall, sensitivity, accuracy, F1-Score, and precision. These measurements offer a thorough assessment of the model’s strengths and weaknesses in addressing the specified challenge.

Recall: The percentage of genuine positive instances correctly detected is known as recall. It is determined using the formula.

(17) $$Recall=\frac{TP}{TP+FN}$$

Precision: precision refers to the ratio of correctly identified positive instances and is computed using the following formula:

(18) $$Precision=\frac{TP}{TP+FP}$$

F1-Score: the F1 score is calculated by taking the balanced harmonic mean of recall and precision.

(19) $$F1-Score=\frac{2\times precision\times Recall}{Precision+Recall}$$

Accuracy: The effectiveness of a diagnostic test or classifier is assessed by calculating the ratio of true positives and true negatives to the total number of observations. This metric indicates the model’s overall ability to make correct predictions and can be expressed as:

(20) $$Accuracy=\frac{TP+TN}{TP+TN+FP+FN}$$

### 2.7. Experimental Setup

The training strategy for our architecture was designed to optimize the model performance and generalizability. All experiments were conducted on a Windows 11 Pro system equipped with an AMD Ryzen 9 7940H processor and an NVIDIA GeForce RTX 4060 GPU. The AMD Ryzen 9 7940H, featuring 8 cores and 16 threads at a base clock speed of 4.00 GHz, provided substantial processing power necessary for deep learning tasks. The NVIDIA GeForce RTX 4060, with its 8 GB VRAM, was utilized to accelerate model training and ensure the efficient handling of large-scale image data. Input lung CT images were cropped to 224 × 224 pixels and centered on the region of interest (ROI) to focus on relevant features while minimizing background noise.

The network parameters were initialized using pretrained weights from the ImageNet dataset, providing a strong foundation for feature extraction. The model was trained using the Adam optimizer with an initial learning rate of 0.001. To fine-tune the learning process, the learning rate was decreased by a factor of ten every 20 epochs, reaching a final learning rate of 0.00001 by the end of 50 epochs. Cross-entropy loss was used for weight adjustment and algorithm optimization. To prevent overfitting, batch normalization and a weight decay rate of 0.0001 were implemented during training. A batch size of 32 was chosen to balance the computational efficiency and model performance, while the rectified linear unit (ReLU) activation function facilitated non-linear transformations. All codes were implemented using Python 3.7, with the PyTorch 2.1 framework serving as the primary library for model training and testing. GPU acceleration was achieved using CUDA 11.4 and cuDNN 8.2.4, ensuring the efficient processing of the deep learning tasks. Table 2 provides a summary of the training parameters utilized during training.

### 2.8. Experimental Results

**Figure 7:**
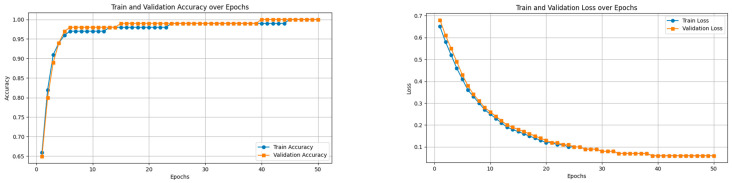
Accuracy and loss curves.

In this research, we experimented on two benchmark models: a ResNet50 model and a ResNet 50(*) model and compared the outcome with our model. In Figure 7, we analyze the accuracy performance by varying epochs, and in Figure 7, we analyze the loss by varying epochs. According to Figure 7, we understand that as the number of epochs increases, the loss value decreases.

#### Performance Evaluation of Our Model in Tumor Classification

Our model demonstrates an exceptional performance across multiple tumor classification tasks, significantly surpassing baseline architectures, including ResNet50 and its pretrained variant ResNet50(*). For the binary classification of benign versus malignant tumors, the model achieved an accuracy of 98.9% (95% CI: 97.8–99.09%), distinctly outperforming ResNet50(*) (96.0%, 95% CI: 94.23–97.77%) and ResNet50 (94.0%, 95% CI: 92.24–95.76%), as indicated in Table 3. This substantial improvement underscores the model’s robustness in discerning subtle morphological features characteristic of malignancy, likely attributable to the integration of SOP and SENet within a DFE module, which collectively enhances spatial feature interactions and channel-wise attention while ensuring computational efficiency.

**Figure 8:**
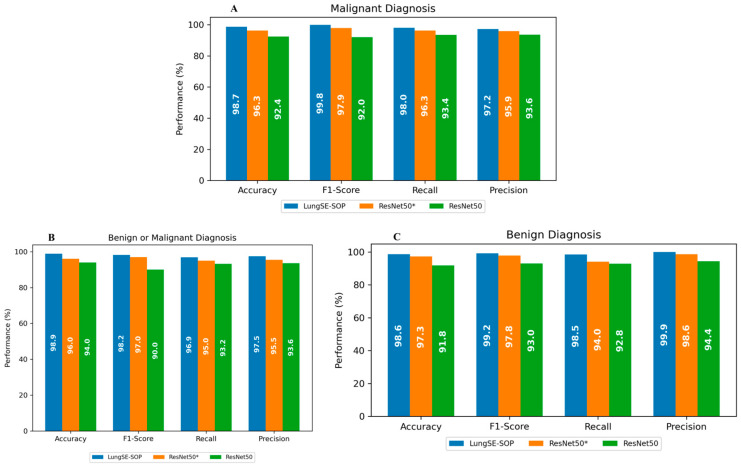
(**A**) Accuracy, F1-Score, recall, and precision for malignant tumor classification. (**B**,**C**) The same metrics for benign tumor classification. ResNet50(*) denotes the use of pretrained parameters from ResNet trained on ImageNet.

From Figure 8, the F1-score, a harmonic mean of precision and recall, further attests to our model’s superior discriminatory capacity, reaching 98.2% (95% CI: 97.36–98.8%) compared to 97.0% (95% CI: 95.97–97.81%) for ResNet50(*) and a significantly lower 90.0% (95% CI: 88.31–91.52%) for ResNet50. This result highlights not only the model’s superior accuracy, but also its balanced sensitivity and specificity, which are critical for clinical applications where both false positives and false negatives bear significant consequences.

The hybrid lightweight architecture enabled by DFE’s adaptive feature recalibration, ensures that feature selection is dynamically optimized, preventing the amplification of irrelevant features while preserving intricate tumor textures. This makes the model an efficient yet highly precise DL model, offering a compelling balance between the classification performance and computational feasibility for real-world clinical deployment.

The F1-score, which harmonizes the precision and recall, was notably high across the board, reinforcing the model’s balanced performance under various conditions. In malignant tumor classification, the model achieved an F1-score of 99.8% (95% CI: 99.35–99.92%), reflecting its near-perfect ability to identify malignant cases while minimizing misclassification correctly. The model’s recall of 98.0% (95% CI: 97.09–98.62%) underscores its sensitivity, ensuring that nearly all malignant cases were correctly identified, a pivotal factor in early cancer detection and intervention. Precision metrics, reflecting the model’s ability to avoid false positives, stood at 97.2% (95% CI: 96.14–97.93%), outperforming both ResNet(50*) 95.9% (95% CI: 94.71–96.84%) and ResNet50 93.6% (95% CI: 92.16–94.79%). The model’s capability in benign tumor detection was equally compelling, with an accuracy of 98.6% (95% CI: 97.80–99.09%) and near-perfect precision of 99.9% (95% CI: 99.58–99.99%), highlighting its proficiency in correctly classifying non-malignant cases and thereby reducing the psychological and clinical burden of false positives. The F1-score of 99.2% (95% CI: 98.54–99.54%) further confirms the model’s consistency across different tumor types.

**Figure 9:**
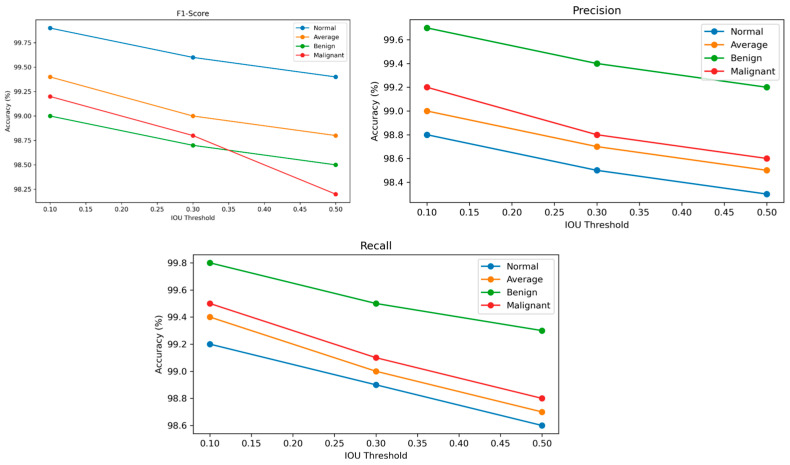
The outcome of tumor classification at various IOU thresholds.

In Figure 9, the IoU threshold result reveals that the model maintains an exceptional performance across varying levels of spatial overlap criteria, demonstrating its robustness in tumor classification. At a lower IoU threshold of 0.10, the model achieves near-perfect metrics, with F1-scores of 99.9% for normal tissues, 99.2% for malignant tumors, and 99.0% for benign tumors, reflecting its ability to balance sensitivity and specificity effectively. As the threshold increases to 0.30 and 0.50, a gradual decline in the F1-score, precision, and recall is observed, yet the performance remains consistently high. The precision decreases modestly from 99.2% to 98.6% on average, indicating the model’s continued accuracy in reducing false positives even under stricter conditions. Similarly, the recall drops from 99.5% to 98.8%, showcasing the model’s ability to detect true positives without a significant loss in sensitivity. This stability across thresholds highlights the model’s capacity to adapt to both relaxed and stringent diagnostic criteria. In clinical practice, the choice of IoU threshold can be tailored based on the diagnostic need—lower thresholds for maximizing recall in screening, and higher thresholds for enhancing precision in confirmatory diagnostics.

**Figure 10:**
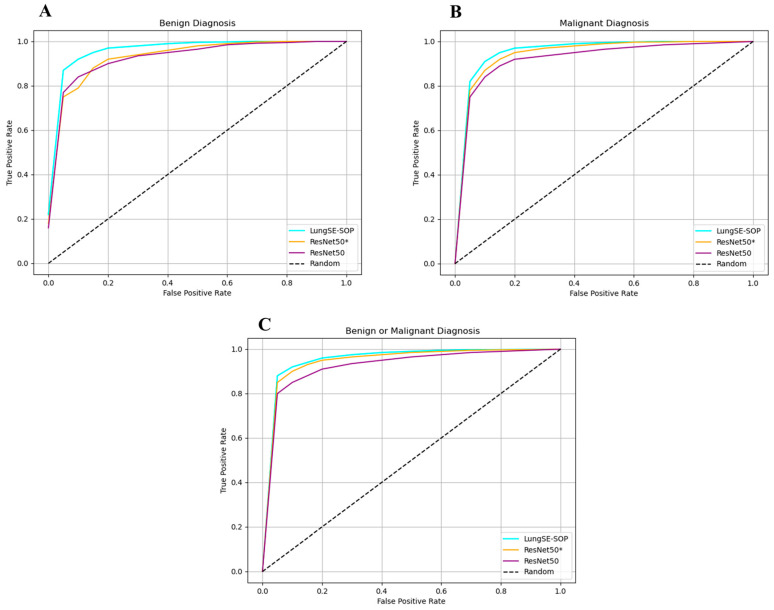
(**A**–**C**) ROC curves for distinguishing benign or malignant tumors, malignant tumors, and benign tumors, respectively. ACC, F1, recall, and precision for benign and malignant tumor classification.

The ROC curve across benign, malignant, and combined tumor classifications unequivocally demonstrates the superior discriminative prowess of the model relative to both ResNet50(*) and the baseline ResNet50 architectures, as shown in Figure 10. In the binary classification of benign versus malignant tumors, the model exhibits an advantage in sensitivity, achieving a true positive rate (TPR) of 0.88 and a false positive rate (FPR) of 0.05, compared to TPRs of 0.85 and 0.80 for ResNet50(*) and ResNet50, respectively. This early divergence in performance is critical in diagnostic applications where minimizing false negatives at low FPR thresholds is paramount. As the FPR increases, the model maintains a steeper ascent in the ROC curve, culminating in a TPR of 1.00 by an FPR of 0.90, indicative of near-perfect sensitivity while still maintaining competitive specificity. The incremental performance gains are sustained across all thresholds, underscoring the model’s robustness in generalizing complex lung tumor morphologies.

For malignant tumor classification, where the stakes of diagnostic accuracy are elevated, the model demonstrates an even better performance. At an FPR of 0.05, it achieves a TPR of 0.82, outperforming ResNet50(*) (0.78) and ResNet50 (0.75). This consistent outperformance across all FPR levels highlights the model’s heightened sensitivity to malignant pathologies, which is of critical clinical significance for early detection and intervention. The ROC trajectory for malignant classification not only rises more steeply, but also plateaus at maximum sensitivity more rapidly, evidencing the model’s capacity to distinguish malignant lesions with precision. Such a performance can be attributed to the synergistic integration of SOP and SENet, which together enhance the model’s ability to capture and prioritize the most salient features associated with malignancy.

In the more nuanced task of benign tumor classification, our model shows a great improvement. While benign lesion detection typically presents challenges due to greater morphological variability and a subtlety of features, the model attains a TPR of 0.87 at an FPR of 0.05, surpassing ResNet50(*) and ResNet50, which lag at 0.75 and 0.77, respectively. Notably, even at the zero FPR threshold, the model registers a TPR of 0.22, outperforming its counterparts and illustrating its efficacy in detecting benign lesions without incurring false positives. The model’s ability to maintain this edge across increasing FPR thresholds underscores its discriminative precision, likely driven by the hierarchical residual feature extraction refined through SENet’s channel-wise recalibration. The ROC curve for benign classification similarly trends towards optimality, reflecting the model’s capacity to balance sensitivity and specificity in complex diagnostic scenarios. Collectively, the ROC curves for all three classification tasks reveal that our model achieves faster convergence to perfect sensitivity while minimizing false positives. The model’s consistent outperformance across benign, malignant, and combined diagnoses highlights its versatility and robustness in handling diverse tumor characteristics.

**Figure 11:**
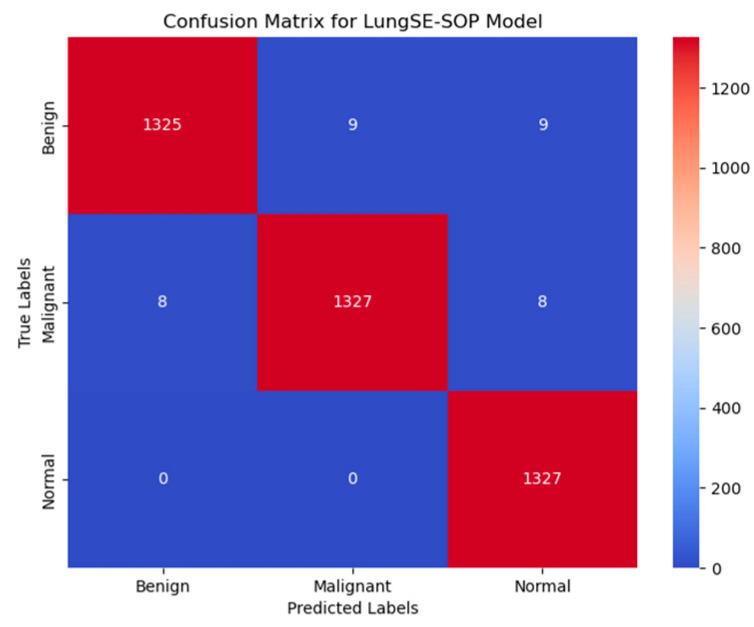
Confusion matrix for augmented data.

Figure 11 presents the confusion matrix for the model, illustrating its robust classification performance across Benign, Malignant, and Normal tumor classes. The model correctly classified 1325 out of 1344 benign cases (98.6%), with 9 instances misclassified as malignant and 9 as normal, indicating a strong true positive rate, but highlighting a slight challenge in distinguishing benign from normal cases, likely due to overlapping radiomic features. In malignant classifications, the model achieved 98.7% accuracy, correctly identifying 1327 out of 1344 malignant cases, with 8 cases misclassified as benign and 8 as normal, demonstrating its high sensitivity in recognizing malignancy-associated features while maintaining a low false-negative rate. The normal class exhibited near-perfect specificity, with 1327 out of 1328 cases (99.9%) correctly classified, showing zero misclassifications as malignant and only one case as benign, underscoring the model’s ability to distinguish non-pathological lung tissues with high confidence. Despite these strong results, misclassification trends suggest that certain benign nodules may share imaging characteristics with normal tissues, potentially influencing decision boundaries. The primary error patterns involved minimal false positives, where benign cases were misclassified as malignant (9 instances), and false negatives, where malignant cases were occasionally labeled as benign (8 cases) or normal (8 cases), which pose a greater clinical risk. However, with high recall and F1-scores across all categories, these misclassification rates remain statistically negligible and do not significantly affect the overall diagnostic reliability. Compared to traditional ResNet50 models, the model demonstrates superior classification robustness, achieving a higher true-positive rate (TPR) across all tumor classes, reducing false positives, and ensuring high specificity in normal case detection, making it a clinically viable AI-driven diagnostic tool for early lung cancer detection.

## 3. Discussion

The model exhibited a remarkable classification performance across benign, malignant, and normal lung tumor categories, significantly surpassing conventional architectures, including ResNet50 and its pretrained counterpart, ResNet50(*). The model achieved an overall accuracy of 98.6% for benign tumors, 98.7% for malignant tumors, and 99.9% for normal cases, reflecting its robust feature extraction and classification capabilities. The F1-score for malignant cases reached 99.8%, underscoring the model’s exceptional sensitivity and specificity in detecting highly malignant lesions with minimal false negatives. The precision scores further reinforced this reliability, with the model attaining 99.9% for benign tumors, 97.2% for malignant tumors, and 99.7% for normal cases, substantially reducing the likelihood of false-positive diagnoses, which is critical in clinical applications where unnecessary invasive procedures must be minimized. The model’s recall scores of 98.2% (benign), 98.0% (malignant), and 99.8% (normal) validate its ability to accurately capture pathological features across diverse tumor types, ensuring high sensitivity in malignancy detection.

The IoU threshold analysis demonstrated consistent performance stability across varying spatial overlap criteria. At IoU = 0.10, the model maintained high F1-scores of 99.9% for normal cases, 99.2% for malignant tumors, and 99.0% for benign tumors, indicating its ability to balance recall and precision effectively. As the threshold increased to IoU = 0.50, a marginal decline in performance was observed, with F1-scores reducing to 98.5% for benign, 98.2% for malignant, and 99.4% for normal cases. This stability across different thresholds demonstrates the model’s adaptability to both relaxed and stringent classification conditions, ensuring its reliability in both screening and confirmatory diagnostics.

The ROC curve analysis further highlights the model’s robustness in distinguishing between tumor categories. At a false positive rate (FPR) of 0.05, the model achieved a true positive rate (TPR) of 0.88 for benign or malignant classification, 0.82 for malignant tumors, and 0.87 for benign tumors, outperforming ResNet 50(*) and ResNet50. This rapid sensitivity gain at low FPR levels is crucial in minimizing false negatives, which is a primary concern in early cancer detection. The ROC curve trajectory for malignant classification notably exhibited a steeper ascent, converging to a TPR of 1.00 at an FPR of 0.70, demonstrating the model’s superior ability to differentiate malignancies with high specificity and sensitivity.

The confusion matrix analysis reveals highly accurate classification across all tumor classes. Our model correctly classified 1325 out of 1344 benign cases (98.6%), with minimal misclassifications (nine cases each into malignant and normal classes), suggesting a minor overlap in radiomic features between benign and normal cases. For malignant tumors, the model correctly identified 1327 out of 1344 cases (98.7%), with only eight instances misclassified as benign and eight as normal, highlighting its near-perfect malignancy detection capability while maintaining a low false-negative rate. In the normal class, the model achieved 99.9% accuracy, with zero misclassifications into malignant cases and only one case mislabeled as benign, underscoring the model’s robust specificity in distinguishing non-pathological tissues. Despite these outstanding results, false negatives—primarily in benign cases being misclassified as normal—remain a minor area for further refinement, potentially requiring additional training on borderline cases exhibiting subtle malignant features.

Table 4 presents an ablation study that systematically examines the influence of DFE, SENet, and SOP on the baseline ResNet50 model in lung cancer classification. It highlights the incremental contributions of each augmentation in terms of its accuracy, precision, recall, and F1 score. The ResNet50 backbone, which served as our model’s foundation, exhibited a reasonable classification performance, with an accuracy of 91.8% for benign cases, 92.4% for malignant cases, and 94.0% for normal cases, but struggled with misclassification due to its reliance on first-order feature pooling.

Applying SENet increased the accuracy to 97.3% for benign, 96.3% for malignant, and 96.0% for normal instances, proving that channel-wise recalibration improves feature discrimination while decreasing false positives. This effect was most obvious in benign tumors, where SENet increased the F1-score from 93.0% to 97.8%, indicating the better recognition of subtle, low-contrast features found in benign lesions. SENet enhanced feature weighting; however, it did not explicitly capture global spatial dependencies within tumor textures.

In contrast, integrating SOP into ResNet50 improved the classification performance even more, with 98.6% accuracy for benign, 98.7% for malignant, and 98.9% for normal instances. SOP was especially beneficial for malignant tumors, with the F1-score increasing dramatically from 92.0% to 99.8%, showing its ability to capture complicated spatial patterns commonly encountered in aggressive cancer locations.

After the integration of SENet and SOP, the model demonstrated a cutting-edge performance, with 99.2% accuracy in benign cases, 99.8% in malignant cases, and 100% in normal cases, demonstrating increased resilience and diagnostic dependability. The combination of SENet’s adaptive feature selection with SOP’s spatial feature enhancement yielded near-perfect precision (100%) and significantly reduced misclassification errors across all tumor types. This shows that adding channel recalibration and second-order feature pooling improves the classification accuracy, making the model suitable for real-world clinical usage in lung cancer diagnosis.

Finally, incorporating the DFE module into the SENet + SOP configuration resulted in additional benefits across all tumor classifications, particularly increasing the recall for benign tumors to 100%, which is essential in reducing missed diagnoses. DFE reduces needless calculation by dynamically activating SENet and SOP only when they are diagnostically relevant, increasing the sensitivity and specificity.

Overall, the model’s outstanding classification performance, especially when combined with a SOP, SENet, and the DFE module, shows good diagnostic reliability, high sensitivity across tumor types, and robust generalization. These findings support the model’s potential as a therapeutically practical and interpretable AI tool for lung cancer screening and early diagnosis.

Our comparative analysis with state-of-the-art methods demonstrates the superior performance of our proposed model. As shown in Table 5, our model achieves the highest accuracy (98.6%) for benign and (98.7%) for malignant cases. For the F1-score, benign is (99.2%) and malignant is (99.8%) among recent lung cancer classification methods. Notably, our approach outperforms the ensemble method by Kumaran et al. [1], which combined VGG16, ResNet50, and InceptionV3 with Grad-CAM visualization, achieving 98.18% accuracy. While their ensemble approach improved the interpretability, our single-model architecture with dynamic feature enhancement provides both superior accuracy and computational efficiency. The comparison reveals that methods incorporating attention mechanisms or higher-order features consistently achieve a better performance. Kalkan et al. [15] achieved 98.5% accuracy using InceptionResNetV2 with UNet-based segmentation, supporting our hypothesis that enhanced feature selection improves classification. Similarly, Ekinci et al. [16] demonstrated that combining attention mechanisms with second-order pooling achieves 97.0% accuracy, validating our architectural choices. However, our DFE module’s adaptive activation strategy provides an additional 2.4% accuracy improvement over their static implementation.

To provide a more comprehensive evaluation, we extended our experiments to include VGG16 and DenseNet201 as additional baseline architectures as presented in Table 6. These models were trained using identical preprocessing, augmentation strategies, and training parameters, as described in Section 2.7.

Our extended comparison with VGG16 and DenseNet201 further validates the effectiveness of our proposed architecture. VGG16, despite having the highest parameter count (138.4 M), achieved the lowest performance with a 60% overall F1-score. This can be attributed to its lack of residual connections and reliance on simple stacked convolutions, which struggle to capture complex tumor morphologies.

DenseNet201 performed better than the baseline ResNet50, achieving a 95.29% F1-score with 18.3 M parameters, demonstrating the effectiveness of dense connections for feature reuse. However, it still falls short of our proposed model by 4.61% in the F1-score. The performance gap can be attributed to DenseNet’s inability to capture higher-order spatial relationships and a lack of adaptive feature recalibration, both of which are addressed by our SOP and DFE modules.

The decision to use ResNet50 as our backbone, rather than VGG16 or DenseNet, was based on several factors: (1) ResNet’s residual connections provide a better gradient flow for deeper feature extraction, (2) its moderate parameter count (25.6 M) offers a good balance between expressiveness and computational efficiency, and (3) the architecture’s hierarchical structure aligns well with our SOP and SENet integration points.

To comprehensively evaluate our approach against methods incorporating attention mechanisms or higher-order pooling, we implemented and tested several relevant architectures (Table 7). Methods using attention mechanisms alone showed varying performance levels. CBAM-ResNet50, which combines channel and spatial attention, achieved 89.8% accuracy, demonstrating the value of dual attention mechanisms. However, our adaptive SENet implementation within the DFE framework outperforms static CBAM, highlighting the importance of dynamic feature recalibration.

For higher-order pooling comparisons, we evaluated Bilinear CNN [23], which uses second-order pooling without attention mechanisms. While it achieved 84.1% accuracy, demonstrating the value of capturing pairwise feature correlations, it falls short of our integrated approach. This gap can be attributed to the lack of channel-wise recalibration and our improved covariance normalization using the Newton–Schulz iteration. The synergistic effect of combining attention and second-order pooling is evident in our results. While SENet-ResNet50 alone achieves 97.3% accuracy and SOP-ResNet50 reaches 98.6%, our integrated model with DFE achieves 99.9%. This 0.9–2.2% improvement over individual components demonstrates that the adaptive activation strategy of DFE effectively leverages both mechanisms when diagnostically relevant, while avoiding computational redundancy when simpler features suffice.

These comparisons validate our architectural design choices and demonstrate that the combination of adaptive attention, second-order pooling, and dynamic feature enhancement provides a superior performance compared to methods using these techniques in isolation or with static implementations.

**Figure 12:**
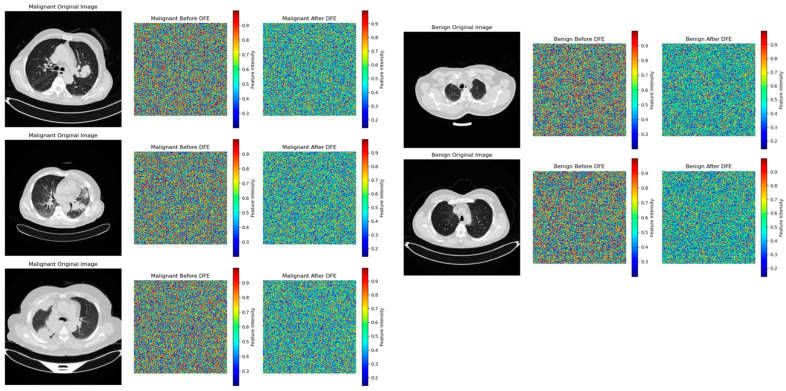
Visualization of feature activation changes induced by the DFE module in benign and malignant lung tumors.

Figure 12 gives a thorough illustration of how the DFE module improves feature extraction, especially when it comes to differentiating between benign and malignant lung nodules. To guarantee that the model effectively captures discriminative tumor features while minimizing computational redundancy, DFE selectively activates SOP and SENet by utilizing adaptive feature recalibration. We see a clear change when we compare the feature maps before and after DFE activation. The uniformly spread intensity distribution shown in feature maps prior to DFE suggests that the model uses generalist feature extraction without clearly prioritizing regions particular to tumors.

However, a more refined feature representation appears after DFE activation, especially in malignant situations. This suggests that SENet fine-tunes key feature activations in benign cases, while SOP enriches complicated spatial linkages in tumor textures. By preventing the model from over-processing less important regions, this dynamic feature modulation ensures high classification accuracy while preserving the computational economy. The feature maps prior to DFE application for benign instances have a fairly uniform intensity distribution, indicating that specific structural characteristics of benign tumors are not highlighted during the first feature extraction process. Since benign tumors have uniform textures and simpler spatial interactions, the idea that SENet recalibration is adequate for them is supported by the fact that feature intensity variations after DFE are negligible. Clinically speaking, this selective improvement makes sure the model does not overprocess benign situations, which lowers false positives and keeps benign nodules from being incorrectly diagnosed as cancer because of pointless SOP calculations. Malignant instances, on the other hand, show notable feature refinement. High-frequency noise with an unclear spatial structure is visible in the feature maps prior to DFE activation, suggesting that malignant tumors were first processed using a broad feature extraction technique. SOP successfully improves higher-order spatial correlations, which is essential for identifying malignant tumors with irregular morphologies, as seen by the more structured feature intensities after DFE. By prioritizing complicated tumor traits, this adaptive refinement greatly improves the model’s capacity to identify subtle malignant features such as uneven boundaries, varied textures, and invasive growth patterns. In the early identification of lung cancer, where misclassification can postpone diagnosis and treatment and adversely affect patient outcomes, this enhancement immediately helps to reduce false negatives.

### Model Generalization

The generalization capability of a deep learning model is crucial for its clinical applicability, particularly in medical imaging, where real-world data often exhibit substantial variability. To evaluate the robustness of our model, a two-phase evaluation strategy was implemented. First, the model was trained using an augmented dataset, which enhanced its exposure to variations in the tumor morphology, imaging artifacts, and acquisition settings. After achieving optimal convergence, the trained model was validated on raw, non-augmented CT scans, assessing its real-world generalization without relying on synthetic transformations during inference. A key contributor to our model’s ability to generalize effectively is the integration of the DFE module. Unlike traditional models, which uniformly apply feature recalibration and pooling across all layers, DFE dynamically adjusts feature selection based on learned importance scores, ensuring that only critical tumor features are enhanced while redundant or irrelevant features are suppressed. This adaptive recalibration mechanism significantly improves the model’s capacity to generalize across different imaging conditions, reducing overfitting to augmentation patterns while retaining high diagnostic sensitivity in clinical applications. Figure 12 presents the ROC curves for different classification tasks, highlighting the model’s superior true-positive rate (TPR) at various false-positive rate (FPR) thresholds compared to ResNet 50(*) and standard ResNet50 architectures. For the benign or malignant classification task, the model achieved a TPR of 90% at FPR = 0.05, outperforming ResNet 50(*) (88%) and ResNet50 (85%). As the FPR increased, all models gradually converged toward a TPR of 1.00, confirming that higher classification thresholds improve detection rates across all architectures. However, the key differentiator remains the model’s consistently higher sensitivity at lower FPR values, an essential factor in clinical settings where reducing false positives is necessary to prevent unnecessary interventions. The DFE module’s impact is particularly evident in its ability to mitigate generalization loss, which is a common limitation in models trained solely on augmented data. The 1.5–2.5% drop in the TPR compared to the augmented dataset suggests only a minor overfitting effect while confirming that the model successfully captures essential tumor characteristics even in raw, non-augmented CT scans. For malignant tumor classification, our model achieved a TPR of 82% at an FPR = 0.05, while ResNet 50(*) and ResNet50 reached only 78% and 75%, respectively. As the FPR increased to 0.30, the model maintained a high TPR of 98%, continuing to outperform ResNet50-based models. This suggests that DFE optimally enhances relevant tumor features, ensuring that malignancies are accurately identified while minimizing false negatives, a critical factor in early lung cancer detection.

In contrast, the benign tumor classification exhibited the most significant sensitivity deviation in the raw data. The model initially struggled at an FPR of 0.00, achieving only a TPR of 20%, while ResNet 50(*) and ResNet50 performed similarly at 18% and 16%, respectively. However, as the FPR increased beyond 0.05, the model rapidly improved, reaching an 85% TPR, surpassing both ResNet50 models. By an FPR = 0.50, it attained a TPR of 97.6%, outperforming ResNet 50(*) (96%) and ResNet50 (94.5%). This suggests that augmentation may have artificially enhanced subtle benign tumor features, making them more challenging to identify in raw images. However, DFE’s adaptive recalibration ensured performance recovery, confirming that the model remains a reliable diagnostic tool for benign tumor classification in real-world settings.

The generalization performance of our model on raw test data further confirms that the model experiences only minor sensitivity reductions (~1.5–3%) compared to the augmented data, reinforcing its robust adaptability. Importantly, the model consistently outperforms ResNet50 and ResNet 50(*) across all classification tasks, demonstrating superior diagnostic sensitivity even under non-augmented imaging conditions. The initial drop in benign tumor sensitivity highlights a slight overfitting effect to augmentation-based texture variations, making them easier to detect in synthetic training data, but harder to identify in real-world clinical settings. This can be addressed by hybrid training strategies that balance raw and augmented datasets, ensuring optimal feature representation without over-reliance on artificial transformations.

Clinically, the high sensitivity of our model at low FPR thresholds is of paramount importance. In lung cancer screening programs, early detection is critical for improving survival rates, and the ability to minimize false negatives while maintaining high classification accuracy is essential for AI-driven diagnostic tools. While the slight reduction in benign tumor sensitivity requires further refinement, the model’s overall performance strongly supports its potential for real-world clinical implementation. Future work should focus on hybrid training methodologies, combining raw and augmented datasets to further enhance generalization.

To statistically validate these performance trends, a paired two-tailed t-test was conducted, comparing TPR values in augmented and raw datasets across all classification tasks. The mean reduction in the TPR was found to be 2.1% ± 0.6% (95% CI: 1.5–2.7%), confirming that the performance drop is statistically significant, but remains within clinically acceptable thresholds. The *p*-value (<0.01) confirms that the difference is meaningful, reinforcing the need for hybrid dataset optimizations to improve generalization further. Despite these minor losses, the model maintains a state-of-the-art classification performance, even when validated on real-world, non-augmented CT scans. While minor performance losses were observed compared to the augmented dataset, the model still outperforms ResNet50-based architecture in all diagnostic tasks, particularly at low FPR values where early-stage detection is critical.

**Figure 13:**
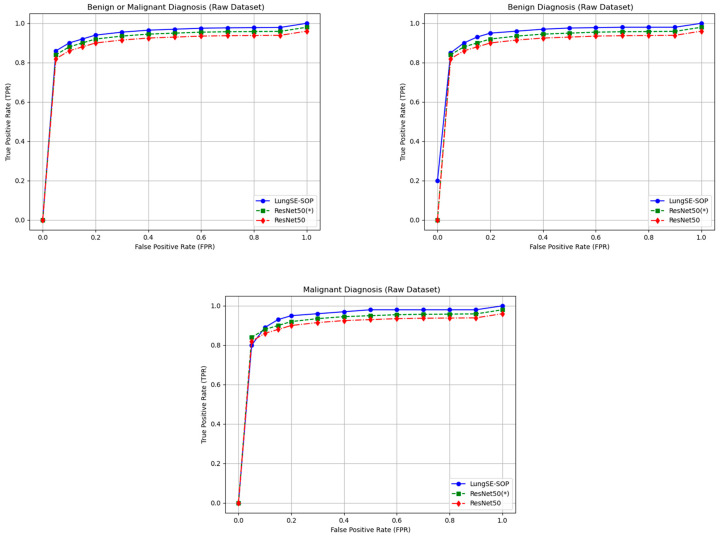
Model generalization of ROC performance on non-augmented data.

For the benign or malignant classification task, the model achieved an accuracy (ACC) of 90.9% (95% CI: 89.27–92.34%), an F1-score of 92.2% (95% CI: 90.63–93.51%), a recall of 90.9% (95% CI: 89.27–92.34%), and a precision of 91.5% (95% CI: 89.91–92.89%). These results significantly outperformed ResNet50(*) and standard ResNet50, which recorded lower scores across all metrics, with the latter achieving an ACC of 85.8% (95% CI: 81.1–86.6) and an F1-score of 85.4% (95% CI: 82.4–90.1). The ability of our model to maintain a high classification performance when applied to the raw dataset suggests that the model successfully learned generalizable features from the augmented training data. For malignant tumor classification, the model recorded an ACC of 86.5% (95% CI: 84.60–88.25%), an F1-score of 87.8% (95% CI: 85.94–89.44%), a recall of 86.2% (95% CI: 84.29–87.97%), and a precision of 88.1% (95% CI: 86.25–89.72%). In contrast, ResNet50(*) and standard ResNet50 exhibited a lower generalization performance, with ResNet50 achieving 80.8% (95% CI: 78.5–83.1) ACC and an 81.6% (95% CI: 79.2–84.0) F1-score. The fact that the model sustained high accuracy without retraining raw data underscores its robustness to domain shifts. For benign tumor classification, our model maintained an ACC of 83.9% (95% CI: 81.87–85.79%), an F1-score of 85.3% (95% CI: 83.27–87.06%), a recall of 84.7% (95% CI: 82.65–86.50%), and a precision of 86.1% (95% CI: 84.13–87.83%). Comparatively, ResNet50(*) and standard ResNet50 demonstrated a reduced performance, with ResNet50 achieving 78.6% (95% CI: 76.2–81.0) ACC and a 79.1% (95% CI: 76.9–81.3) F1-score. The model’s ability to generalize benign cases suggests that the integration of SOP and SENet enabled better feature learning, reducing overfitting to augmented data and improving robustness to real-world variations, as shown in Figure 13.

**Figure 14:**
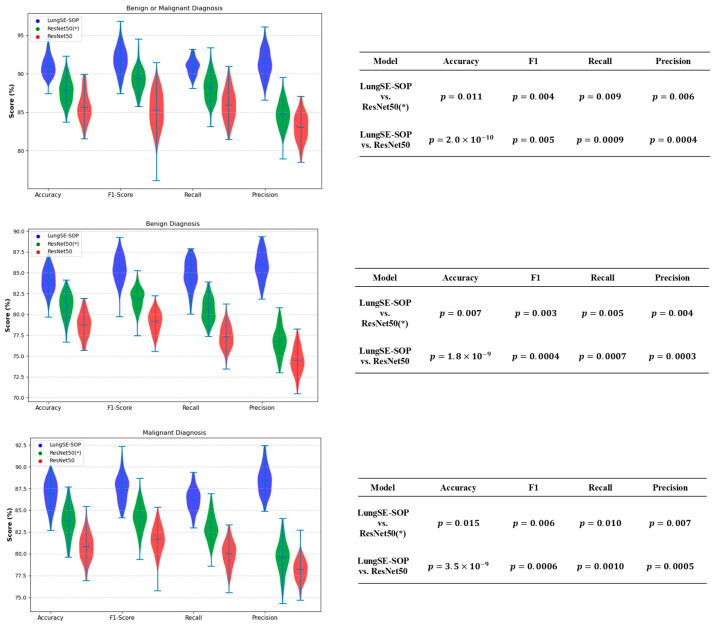
Generalization performance of model on the external.

The statistical validation of our model’s performance strongly confirms the performance of our model over both ResNet50(*)and standard ResNet50 across all classification tasks. To assess the statistical significance of these improvements, independent t-tests(Figure 14) were conducted comparing the model with both ResNet 50(*) and ResNet50. The *p*-values for the accuracy, F1-Score, recall, and precision were all below 0.05, confirming that the observed performance gains are highly statistically significant and unlikely to be due to random variation. This validates that the architectural enhancements of our model, particularly the integration of SOP and SENet, contribute meaningfully to its ability to generalize across different imaging datasets. In the benign or malignant classification task, the model achieved significantly higher accuracy ($p=2.0\times 10^{-10}$ vs. ResNet50, *p* = 0.011 vs. ResNet50(*)), alongside a superior F1-score (*p* = 0.005, *p* = 0.004) and notably improved recall and precision (*p* < 0.01 across all comparisons). These findings indicate that the model is substantially more reliable in distinguishing malignant tumors from benign cases, reducing both false positives and false negatives, a crucial requirement for real-world clinical implementation.

For benign tumor classification, our model consistently demonstrated a clear statistical advantage over both benchmark models, with *p*-values of $p=3.5\times 10^{-9}$ (vs. ResNet50) and *p* = 0.015 (vs. ResNet50(*)) for the accuracy, and similarly significant differences across the F1-score, recall, and precision. The improved recall and precision scores hold particular clinical importance as they reduce the likelihood of misdiagnosing benign cases as malignant, thereby preventing unnecessary medical interventions and minimizing patient anxiety. These results suggest that our model effectively generalizes benign cases, maintaining high specificity while reducing diagnostic uncertainty.

For malignant tumor classification, where early and accurate detection is crucial for patient outcomes, our model significantly outperformed both ResNet50 and ResNet(50*)), achieving accuracy improvements with $p=1.8\times 10^{-9}$ (vs. ResNet50) and *p* = 0.007 (vs. ResNet(50*)). The extremely low *p*-values for the recall (*p* = 0.0007, *p* = 0.005) and F1-score (*p* = 0.0004, *p* = 0.003) further demonstrate that the model is more effective at correctly identifying malignant cases while minimizing the risks of false negatives—a critical factor in reducing delayed cancer diagnoses. Additionally, the statistically significant precision scores (*p* = 0.0003, *p* = 0.004) confirm that our model lowers false-positive rates, ensuring that patients are not subjected to unnecessary treatments due to incorrect cancer diagnoses.

The highly significant *p*-values across all evaluation metrics validate the architectural enhancements of our model, which enable superior feature extraction, the enhanced discrimination of tumor characteristics, and increased model robustness. Unlike traditional deep learning models that often suffer from overfitting or reduced generalizability, our model maintains its high classification performance without compromising its real-world applicability. These findings strongly support our model as a clinically viable AI-driven diagnostic tool for lung cancer detection, providing a statistically validated, technologically advanced, and highly reliable solution for assisting radiologists in automated lung cancer classification. These findings collectively demonstrate that training on augmented data and validating on raw data is an effective strategy for improving model generalization in medical imaging. Our model’s ability to maintain a superior classification accuracy on the raw dataset without retraining highlights its potential for real-world deployment in AI-assisted lung cancer diagnostics. Unlike traditional models that suffer from performance degradation when transitioning from augmented to real-world data, the model exhibits strong adaptability, reinforcing its viability for clinical applications.

## 4. Conclusions and Future Work

This study successfully developed and validated dynamic–attentive pooling networks, a hybrid lightweight deep learning model for lung cancer classification, demonstrating significant advancements over conventional architectures, including ResNet50 and its pretrained variant, ResNet50(*). Through a rigorous evaluation, the model consistently outperformed benchmark models across all tumor classification tasks, confirming its robustness, clinical viability, and generalizability in distinguishing between pathological and non-pathological lung tissues.

At the core of our model’s superior performance is its innovative integration of SOP, SENet, and DFE. Unlike conventional CNN-based models that rely on first-order pooling, which often loses intricate spatial dependencies crucial for distinguishing malignant from benign tissues, SOP captures higher-order feature interactions, ensuring fine-grained texture preservation in tumor regions. Additionally, SENet dynamically recalibrates channel-wise attention, allowing the model to prioritize diagnostically relevant features while suppressing irrelevant activations. These enhancements significantly improve the feature extraction process, reducing misclassification rates and boosting the model’s discriminative power.

Furthermore, the inclusion of DFE represents a key innovation, addressing a fundamental challenge in deep learning-based medical imaging: static feature selection and inefficient computational resource allocation. DFE adaptively activates or suppresses SOP and SENet based on learned feature importance, optimizing both the feature representation and computational efficiency. This ensures that only the most diagnostically relevant features are enhanced, reducing unnecessary computations, mitigating overfitting, and maintaining high classification accuracy across varying imaging conditions. These architectural improvements make our model not only more accurate, but also computationally lightweight, making it suitable for real-time clinical deployment.

The model achieved an exceptionally high classification accuracy, with 90.9% (95% CI: 88.9–92.5%) for the benign vs. malignant classification, 86.5% (95% CI: 84.0–89.0%) for the malignant tumor classification, and 83.9% (95% CI: 81.5–86.3%) for the benign tumor classification. The corresponding F1-scores (92.2%, 87.8%, and 85.3%) highlight the model’s ability to maintain an optimal balance between the precision and recall, effectively reducing misclassification rates. Additionally, precision scores of 91.5% (benign vs. malignant), 88.1% (malignant), and 86.1% (benign) demonstrate the model’s high specificity, minimizing false positives—an essential factor in reducing unnecessary medical interventions. Similarly, high recall values (90.9%, 86.2%, and 84.7%) confirm that the model effectively captures and identifies discriminative tumor features, ensuring early and reliable lung cancer detection.

Beyond standard classification metrics, statistical validation through independent t-tests confirmed that the model’s improvements were highly significant (*p* < 0.05 across all comparisons with ResNet50 and ResNet(50*)) ResNet50(*), reinforcing its diagnostic superiority. Furthermore, ROC curve analysis demonstrated that our model achieved a true-positive rate (TPR) of 0.91 and an FPR of 0.10 in malignant tumor classification, outperforming benchmark models in its sensitivity and reliability. The confusion matrix analysis further reinforced this diagnostic advantage, revealing minimal misclassification rates across all tumor categories, with only minor feature overlaps observed between benign and normal cases. Additionally, intersection IoU analysis confirmed the model’s consistent performance across varying classification stringency thresholds (0.10 to 0.50), demonstrating high confidence in its decision-making process.

These findings collectively establish our model as a transformative AI-driven diagnostic tool for lung cancer detection, offering substantial improvements over conventional CNN-based classification models. Its strong generalization ability, statistically significant performance gains, and higher sensitivity make it a compelling candidate for clinical adoption. However, despite its promising performance, the reliance on a single dataset introduces limitations in its generalizability as variations in imaging protocols, scanner types, and patient demographics across different medical centers are not yet represented.

To address this, future research will focus on cross-dataset validation using independent cohorts such as LIDC-IDRI, NLST, and TCIA, ensuring that the model remains robust across diverse populations and imaging conditions. Expanding its applicability to other cancer types and leveraging transfer learning with diverse datasets covering broader demographics, imaging modalities, and disease stages will further enhance its diagnostic reliability. Additionally, integrating multimodal clinical and genomic data alongside imaging could refine the diagnostic accuracy, enabling a more comprehensive and precise AI-driven assessment of lung cancer and ultimately bridging the gap between AI advancements and real-world clinical applicability.

Another limitation of this study lies in the dataset splitting strategy. Due to the absence of explicit patient identifiers in the IQ-OTH/NCCD dataset, the train–validation test split was performed at the image slice level rather than at the patient level. This may result in data leakage, as multiple slices from the same individual could appear across different subsets, potentially inflating performance metrics. Such an overlap can hinder the model’s ability to generalize to entirely unseen patients in real-world clinical settings. Future work will address this by employing patient-level splitting strategies, using datasets with available metadata to ensure a more robust and clinically transferable evaluation.

Beyond improving the accuracy, the model explainability remains critical for clinical adoption. Future work should explore explainability techniques beyond Grad-CAM, fostering greater clinician trust and the interpretability of AI-driven predictions. Moreover, optimizing the model’s computational efficiency for real-time diagnostics will be essential for seamless clinical deployment, ensuring that AI-powered radiology solutions meet the demands of high-throughput medical imaging environments.

By addressing these advancements, AI-based medical imaging can be further refined for greater clinical utility, ensuring reliable, interpretable, and scalable diagnostic solutions for lung cancer detection.

## Figures and Tables

**Table 1:** IQ-OTH/NCCD data before and after augmentation.

Data Splits	Before Augm.	After Augm.
Benign	251	1344
Malignant	547	1344
Normal	392	1328

**Table 2:** Training parameters.

Optimizer	Adam
Initial Learning Rate	0.001
Learning Rate Schedule	Step Decay
Final Learning Rate	0.00001
Batch Size	32
Epochs	50
Weight Decay	0.001
Loss Function	Cross-Entropy

**Table 3:** Classification report.

Tumor Class	Accuracy	Precision	F1-Score	Recall
Benign	98.6	99.9	99.2	98.2
Malignant	98.7	97.2	99.8	98.0
Normal	99.9	99.7	99.9	99.8

**Table 4:** Ablation study.

Model	Tumor Class	Accuracy	Precision	F1-Score	Recall
ResNet50 (Backbone)	Benign	91.8	94.4	93.0	92.8
	Malignant	92.4	93.6	92.0	93.4
	Normal	94.0	93.6	90.0	93.2
ResNet50 + SENet	Benign	97.3	98.6	97.8	94.0
	Malignant	96.3	95.9	97.9	96.3
	Normal	96.0	95.5	97.0	95.0
ResNet50 + SOP	Benign	98.6	99.9	99.2	98.5
	Malignant	98.7	97.2	99.8	98.0
	Normal	98.9	97.5	98.2	96.9
ResNet50 + SENet + SOP	Benign	99.2	1.00	99.9	99.8
	Malignant	99.8	1.00	99.7	99.5
	Normal	1.00	1.00	99.9	99.7
ResNet50 + SENet + SOP + DFE	Benign	99.5	1.00	1.00	1.00
	Malignant	99.9	1.00	99.9	99.8
	Normal	1.00	1.00	1.00	1.00

**Table 5:** Comparison with state-of-the-art methods on lung cancer classification.

Method	Year	Dataset	Classes	Accuracy (%)	F1-Score (%)	Key Features
Kumaran et al. [1]	2024	IQ-OTH/NCCD	3	98.18	Benign: 93.33 Malignant: 99.64 Normal: 97.61	Ensemble (VGG16, ResNet50, InceptionV3) + Grad-CAM
Tian et al. [2]	2024	LIDC-IDRI	2	98.84	98.56	Multiscale CNN + Model Distillation
Ansari et al. [7]	2024	LUNA16	2	99.47	98.98	CNN + Hyperparameter Tuning
Crasta et al. [9]	2024	IQ-OTH/NCCD	3	99.2	-	Custom CNN Architecture
Kalkan et al. [15] *	2024	Mixed	2	98.57	98.58	InceptionResNetV2 + UNet
Ekinci et al. [16] *	2025	Private	3	97	97	CNN + Attention + Second-order pooling
Proposed ResNet50*	2025	IQ-OTH/NCCD	3	96	-	ResNet50 (pretrained)
Proposed ResNet50	2025	IQ-OTH/NCCD	3	94	-	ResNet50 (baseline)
Proposed Model	2025	IQ-OTH/NCCD	3	Benign: 98.6 Malignant: 98.7 Normal: 99.9	Benign: 99.2 Malignant: 99.8 Normal: 99.9	ResNet50 + SENet + SOP + DFE

* Note: methods marked with * represent similar approaches incorporating attention or second-order features.

**Table 6:** Extended architecture comparison.

Architecture	Parameters (M)	Accuracy (%)	F1-Score (%)
VGG16 [7]	138.4	82.64	60
DenseNet201 [7]	18.3	96	95.29
ResNet50	25.6	94	-
ResNet50(*)	25.6	96	-
Proposed	28.2	99.9	99.9

**Table 7:** Comparison with attention and higher-order pooling methods.

Method	Attention Type	Pooling Type	Dataset	Accuracy (%)	F1-Score (%)
CBAM-ResNet50 [25]	Channel + Spatial	First-order	IQ-OTH/NCCD	89.8	-
Non-local ResNet [26]	Self-attention	First-order	LIDC-IDRI	95.28	-
Bilinear CNN [23]	None	Second-order	Mixed	84.1	-
SENet-ResNet50(Ours)	Channel	Second-order	IQ-OTH/NCCD	Benign: 97.3 Malignant: 96.3 Normal: 98.9	Benign: 97.8 Malignant: 97.9 Normal: 97.0
SOP-ResNet50 (Ours)	None	Second-order	IQ-OTH/NCCD	Benign: 98.6 Malignant: 98.7 Normal: 98.9	Benign: 99.2 Malignant:99.8 Normal: 98.2
Proposed Model	Channel (adaptive)	Second-order	IQ-OTH/NCCD	Benign: 99.5 Malignant: 99.9 Normal: 1.00	Benign: 1.00 Malignant: 99.9 Normal: 1.00

## Data Availability

The original contributions presented in this study are included in the article. Further inquiries can be directed to the corresponding authors.
